# Supplementary material for: The impact of obesity on static and proactive balance and gait patterns in sarcopenic older adults: an analytical cross-sectional investigation
Source: PeerJ. 2023 Nov 23;11:e16428. doi: 10.7717/peerj.16428 (PMC10676719; doi:10.7717/peerj.16428)
Supplement: Supplemental Information 2 [file peerj-11-16428-s002.pdf]

15 June 2023

To Whom It May Concern:

RE: Effects of physical activity program on body composition, physical performance and neuromuscular strategies during walking in older adults with sarcopenic obesity.

As project manager for the Pan African Clinical Trial Registry (pactr.samrc.ac.za) database, it is my pleasure to inform you that your application to our registry has been accepted. Your unique identification number for the registry is **PACTR202306912191110**.

Please be advised that your trial is registered under an initiative within our system that allow us to capture data of trials that are already in progress or completed. As such, your trial registration may not adhere to the mandates set forth by the International Committee of Medical Journal Editors for registration requirements, and it is your duty to be transparent to any journal that may ask about the retrospective status of your registration.

Please note you are responsible for updating your trial, or for informing us of changes to your trial. Additionally, please provide us with copies of your ethical clearance letters as we must have these on file (via email or post or by uploading online) at your earliest convenience if you have not already done so.

Please do not hesitate to contact us at +27 21 938 0835 or email pactradmin@mrc.ac.za should you have any questions.

Yours faithfully,

PACTR Admin
pactr.samrc.ac.za
+27 021 938 0835
