# Supplementary material for: The impact of obesity on static and proactive balance and gait patterns in sarcopenic older adults: an analytical cross-sectional investigation
Source: PeerJ. 2023 Nov 23;11:e16428. doi: 10.7717/peerj.16428 (PMC10676719; doi:10.7717/peerj.16428)
Supplement: Supplemental Information 3 [file peerj-11-16428-s003.doc]

STROBE Statement—Checklist of items that should be included in reports of ***cross-sectional studies***

|  | Item No | Recommendation | Relevant text from manuscript |
| --- | --- | --- | --- |
| **Title and abstract** | 1 | (*a*) Indicate the study’s design with a commonly used term in the title or the abstract | The interplay of obesity on static and proactive balance and gait patterns in sarcopenic older adults: An analytical coss-sectional investigation |
|  |  | (*b*) Provide in the abstract an informative and balanced summary of what was done and what was found | This study sought to define the impact of obesity on both steady-state and proactive balance, as well as gait characteristics in seniors diagnosed with sarcopenic obesity (SO). The secondary aim was to ascertain the relationship between specific anthropometric measures and balance and gait parameters in this demographic.  We recruited 42 participants, classifying them based on body mass index (BMI) into control (CG; n = 22) and sarcopenic obese (SOG; n = 20) groups. Various assessments were conducted to capture a spectrum of metrics, ranging from body composition variables to balance and gait dynamics, using tests such as the Romberg, Functional Reach, Timed Up and Go, and the 10-meter walking test.  Our analyses illustrated pronounced differences between the two groups. The SOG group, when compared to the CG, revealed compromised results in both the Functional Reach and several gait metrics. Notably, regression analyses pinpointed both body mass and lean body mass (LBM) as key determinants, with LBM accounting for a substantial portion of the observed variations in balance and gait attributes. |
| Introduction | | |  |
| Background/rationale | 2 | Explain the scientific background and rationale for the investigation being reported | 1. **Scientific Background:**   **Age-related Obesity and Sarcopenic Obesity:** The introduction starts by highlighting the increasing global prevalence of obesity in the senior population and introduces the condition of sarcopenic obesity (SO).  Impacts of SO: The introduction indicates the detrimental health effects of SO, like functional impairments and injury risks.  **Importance of Walking in Seniors with SO:** Walking, a routine activity, is essential for disease prevention and autonomy preservation in older adults with SO.  **Age-related Muscle Deterioration:** Factors contributing to muscle decline with age are detailed, from hormonal imbalances to reduced physical activity, which compromises functional abilities, postural stability, and walking proficiency.  **Sarcopenia and Walking Stability:** The introduction underscores how sarcopenia affects older adults' walking stability, leading to reductions in gait speed, balance, and stride length.  **Effects of Obesity on Balance and Gait:** The introduction delves into the recognized effects of adult obesity on balance and its consequences on dynamic activities, such as walking. It also acknowledges existing research on how obesity affects gait metrics in the general population.   1. **Rationale for the Investigation:**   **Contested Effects of Obesity on Sarcopenic Older Adults:** The introduction indicates a disparity in research findings regarding obesity's influence on gait and physical functionality in older adults, especially those with sarcopenia. This highlights the need for clearer categorization and in-depth study of SO.  **Lack of Research on SO in Older Adults:** While some studies have explored the effects of obesity on balance and gait, few have specifically targeted older adults with SO, highlighting a gap in the literature.  Anthropometric Features and Balance in Obese Individuals: Research has identified a correlation between anthropometric features and balance in obese individuals, but there's limited knowledge when it comes to SO in older adults.  **Uncertainty about Muscle Mass vs. Body Fat's Impact:** The introduction stresses the importance of discerning whether reduced muscle mass or increased body fat primarily affects functional capacities in elderly individuals with SO. |
| Objectives | 3 | State specific objectives, including any prespecified hypotheses | **Primary Objective:** The study aims to understand the role of obesity in determining steady-state and proactive balance and gait characteristics in older adults with SO.  **Secondary Objective:** The study seeks to uncover the relationships between anthropometric measures and balance and gait metrics in the same demographic. |
| Methods | | |  |
| Study design | 4 | Present key elements of study design early in the paper | This research adhered to an analytical cross-sectional design that unfolded over 4 months. The study had three significant phases: recruitment, screening, and experimental testing, culminating in a comprehensive 2-hour experimental protocol. This process was designed to gather in-depth data related to the health status, anthropometric measurements, static steady-state balance, proactive balance, and gait characteristics of the participants. |
| Setting | 5 | Describe the setting, locations, and relevant dates, including periods of recruitment, exposure, follow-up, and data collection | The study began its recruitment process between January and March 2022. The selected participants underwent an experimental testing phase lasting 9 weeks. The research was conducted in various regional obesity care centers, with the experimental phase being carried out in a specialized clinical examination room. |
| Participants | 6 | (*a*) Give the eligibility criteria, and the sources and methods of selection of participants | The recruitment pool comprised 72 volunteers from regional obesity care centers. The eligibility criteria included a handgrip force under 17 N, a gait speed below 1.0 m/s, an age of 65 years or older, ability for verbal communication, and physical independence. Exclusion criteria comprised severe neurological or cognitive impairments, significant cardiovascular diseases, significant musculoskeletal issues, other chronic diseases, medications that might impact assessments, or a Montreal Cognitive Assessment (MoCA) score below 26. After applying these criteria, 45 candidates qualified. However, due to adherence challenges, the final participant count was 42. |
| Variables | 7 | Clearly define all outcomes, exposures, predictors, potential confounders, and effect modifiers. Give diagnostic criteria, if applicable | **Outcomes:**  **Anthropometric Measurements:** Objective data such as height, waist circumference, hip circumference, body mass, percentage of body fat mass, fat body mass, and lean body mass.  **Static Steady-State Balance:** Measured by the Romberg Test (ROM), with the outcome being the standing time in seconds without any stability loss incidents.  **Proactive Balance:** Evaluated using the Functional Reach Test (FRT) and the Timed Up and Go Test (TUG). Outcomes were the maximal reach distance in centimeters (for FRT) and the best time achieved in seconds to complete the TUG test.  **10-meter Walking Test:** The primary outcome was the maximum gait speed, with secondary outcomes including spatiotemporal gait parameters such as cadence, speed, stride length, stride time, and various phases of the gait cycle.  **Exposures:**  The exposure in this study pertains to the categorization of participants into two groups based on their body mass index (BMI) – the control group (CG) and the sarcopenic obese group (SOG).  **Predictors:**  - BMI (kg/m²)  - Handgrip force (N)  - Gait speed (m/s)  **Potential Confounders:**  - Physical activity level  - Severe neurological or cognitive impairments  - Significant cardiovascular diseases  - Major musculoskeletal deformities or injuries  - Other chronic diseases  - Medications that could potentially affect the outcomes of the assessments  -A Montreal Cognitive Assessment (MoCA) score below 26  -cardiovascular diseases, nueurological or cognitive impairment  **Diagnostic Criteria**  **- Sarcopenic Obesity:** Likely determined by BMI and potentially by muscle mass or strength measurements, though specific criteria were not given.  **- Cognitive Status:** Assessed using the Montreal Cognitive Assessment (MoCA), with a score below 26 being a criterion for exclusion.  **- Health Status:** This was assessed using the "Health Questionnaire and Medical Records of the Participants." This tool would provide comprehensive insights into the participants' overall health, capturing details of their medical history, current health status, and any chronic conditions or medications that might affect their participation in the study. |
| Data sources/ measurement | 8* | For each variable of interest, give sources of data and details of methods of assessment (measurement). Describe comparability of assessment methods if there is more than one group | **Anthropometric parameters**  Source: Data collected during the recruitment phase.  Method of Assessment: Age was directly reported by the participants. BMI was calculated as weight (kg) divided by height (m) squared.  Comparability: Same method was used for both the CG and SOG.  **Handgrip Force**  Source: Physical evaluations during the screening phase.  Method of Assessment: Measurement tools or instruments used to measure force and speed were not mentioned.  Comparability: Same instruments and protocols applied to both groups.  **Physical Activity Level**  Source: Ricci and Gagnon scales.  Method of Assessment: Participants completed the scales, which assess levels of physical activity.  Comparability: Both groups underwent the same assessment via the scales.  **Health Status**  Source: Health Questionnaire and Medical Records of the Participants.  Method of Assessment: Participants filled out the questionnaire and provided relevant medical records.  Comparability: Both groups were assessed using the same health questionnaire and method of gathering medical records.  **Anthropometric Measurements**  Source: Physical examination.  Method of Assessment: Measurements like height, waist, and hip circumference were taken using a tape measure. Body mass and body fat percentage were evaluated using an impedance-meter.  Comparability: Uniform method applied across both groups in a specialized clinical examination room.  **Static Steady-State Balance**  Source: Romberg Test.  Method of Assessment: Participants were instructed to follow a specific protocol for the test, with standing time in seconds being recorded.  Comparability: Same testing conditions and protocol were applied to both groups.  **Proactive Balance**  Source: Functional Reach Test (FRT) and Timed Up and Go Test (TUG).  Method of Assessment: Specific instructions were provided for each test, and measurements like reach distance in centimeters and time in seconds were recorded.  Comparability: Both groups underwent the same tests under the same conditions.  **Gait Analysis**  Source: 10-meter walking test.  Method of Assessment: Participants walked a 20-meter corridor, with data from the 5th to 15th meters being specifically captured using a wireless inertial sensor system. Specific spatiotemporal parameters of the gait were recorded.  Comparability: Both groups were subjected to the same testing method, equipment, and conditions.  **Cognitive Status**  Source: Montreal Cognitive Assessment (MoCA).  Method of Assessment: Participants completed the MoCA test.  Comparability: The same version and method of administering the MoCA were applied to both groups. |
| Bias | 9 | Describe any efforts to address potential sources of bias | Efforts to address potential sources of bias in the study include:  **- Rigorous Selection Criteria:** The study utilized specific inclusion and exclusion criteria to ensure a consistent and homogenous sample, reducing selection bias  **- Uniform Testing Conditions:** To avoid measurement or instrument bias, all tests were administered in a specialized clinical examination room by a singular assessor. This ensures consistency in the conditions and methods of assessment.  **- Standardized Instructions:** Providing standardized verbal directives to participants during tests ensures that everyone receives the same guidance, minimizing the potential for instruction or participant misunderstanding bias.  **- Use of Validated Instruments and Scales:** Employing recognized and validated assessment toolsensures the reliability of the measurements.  **- Power Analysis:** The study's sample size was rigorously determined using G*Power, ensuring adequate power to detect an effect, minimizing the chance of type II errors (false negatives). |
| Study size | 10 | Explain how the study size was arrived at | To determine the optimal sample size for our study, we utilized the G*Power software (version 3.1.9.4). The parameters were set with the aim of controlling Type I error at an alpha level of 0.05 and Type II error at a beta level of 0.60. This is an acknowledgment of the risks associated with falsely rejecting a true null hypothesis (Type I error) and falsely accepting a false null hypothesis (Type II error).  Furthermore, based on prior research and the objectives of our study, an anticipated moderate effect size of r = 0.35 was considered. Effect size gives an indication of the magnitude of differences we are trying to detect, and in this case, a moderate effect was deemed significant for the study's goals.  Given these parameters (alpha, beta, and the effect size), the power analysis indicated a requirement for a minimum of 40 participants to be statistically confident in the findings.  Embarking on the recruitment phase between January and March 2022, we managed to attract the interest of 72 volunteers from various regional obesity care centers. However, ensuring the integrity and quality of the study, we had a strict adherence to our pre-established inclusion and exclusion criteria. This rigorous selection process meant that out of the initial 72, only 45 candidates were deemed eligible.  Nevertheless, as with many longitudinal studies, challenges related to participant adherence to the regimen led to a dropout of three individuals. This brought our final sample size to 42 participants, slightly exceeding our minimum requirement and thus fortifying the study's potential for statistical significance. |
| Quantitative variables | 11 | Explain how quantitative variables were handled in the analyses. If applicable, describe which groupings were chosen and why | Quantitative variables in our study were primarily derived from the comprehensive experimental protocol which encompassed assessments of participants' health status, anthropometric measurements, static steady-state balance, proactive balance, and the 10-meter walking test. These variables were obtained through standardized procedures, ensuring the reliability and consistency of the data.  **Handling of Variables:**  Continuous variables like height, waist and hip circumference, body mass (BM), percentage of body fat mass (FBM, %), standing time in seconds, maximal reach distance in centimeters, time achieved in the Timed Up and Go Test, and various spatiotemporal parameters from the 10-meter walking test were all captured in their respective units.  The normality of these variables was checked using the Shapiro-Wilk test, ensuring that the data distribution was appropriate for subsequent parametric statistical analyses.  Homogeneity of variance, which assesses if the variance across groups is similar, was confirmed using the Levene's test.  **Groupings:**  Based on their body mass index (BMI, kg/m²), participants were allocated into two distinct groups. The rationale behind this classification was to compare the effects of different BMI levels on the study's outcomes. The groups were:  Control Group (CG; n = 22; age = 81.1 ± 4.0 years; BMI = 24.9 ± 0.6 kg/m²)  Sarcopenic Obese Group (SOG; n = 20; age = 77.7 ± 2.9 years; BMI = 34.5 ± 3.2 kg/m²)  The allocation into these specific groups allowed for a comparison between typical aging individuals (control group) and those who are both aging and sarcopenic obese (SOG). This distinction aimed to isolate the effects of sarcopenic obesity from the normal aging process.  **Analysis:**  To discern the differences between these groups for all parameters, a one-way ANOVA was conducted. This statistical method is suitable for comparing means across multiple groups.  Given the multiple tests, a Bonferroni post-hoc correction was applied to account for the risk of Type I error inflation.  Furthermore, to ascertain the relationship between anthropometric measures (like BMI) and gait/balance parameters, a Spearman correlation analysis was employed. This non-parametric method determines the strength and direction of the monotonic relationship between two variables. |
| Statistical methods | 12 | (*a*) Describe all statistical methods, including those used to control for confounding | The statistical analysis was conducted using Jamovi (Software 2.3, Sydney, Australia). The normality and homogeneity of variance were assessed using the Shapiro-Wilk and Levene tests, respectively. It was confirmed that all parameters met the assumptions of normality and homogeneity of variance. To analyze the differences among groups, a one-way ANOVA was performed for all parameters, considering the factor of obesity. Bonferroni post-hoc procedures were applied to account for multiple comparisons. Additionally, we employed a Spearman correlation analysis to elucidate the association between anthropometric measures and gait/balance parameters. The results were expressed as means (standard deviation). The significance level was set at p < 0.05. |
|  |  | (*b*) Describe any methods used to examine subgroups and interactions | Not applicable |
|  |  | (*c*) Explain how missing data were addressed | Not applicable |
|  |  | (*d*) If applicable, describe analytical methods taking account of sampling strategy | Not applicable |
|  |  | (*e*) Describe any sensitivity analyses | Not applicable |
| Results | | |  |
| Participants | 13* | (a) Report numbers of individuals at each stage of study—eg numbers potentially eligible, examined for eligibility, confirmed eligible, included in the study, completing follow-up, and analysed | **Potentially Eligible:**  A total of 72 volunteers were initially garnered from various regional obesity care centers. These are the individuals who showed initial interest and were potentially eligible to participate in the study.  **Examined for Eligibility:**  All 72 volunteers were examined for eligibility using a series of criteria, including physical tests, questionnaires, and the MoCA test.  **Confirmed Eligible:**  After stringent adherence to the pre-established inclusion and exclusion criteria, 45 candidates were found to be eligible for the study. This means that out of the initial 72, only 45 matched the strict criteria and were confirmed eligible.  **Included in the Study:**  Even after confirming eligibility, not all candidates made it to the actual study phase due to various reasons. 3 individuals dropped out due to challenges related to adhering to the study's regimen. Thus, out of the 45 eligible candidates, 42 were included and began the study.  **Analysed:**  The final analysis was conducted on the data from all 42 participants. These analyses included the application of the one-way ANOVA, Spearman correlation, among others, to interpret the gathered data and draw meaningful conclusions. |
|  |  | (b) Give reasons for non-participation at each stage | **Potentially Eligible to Examined for Eligibility:**  All 72 volunteers who expressed interest were moved forward to the eligibility examination phase. Therefore, there were no dropouts or reasons for non-participation at this stage.  **Examined for Eligibility to Confirmed Eligible:**  Out of the 72 examined, 27 were deemed ineligible. The reasons for their ineligibility included:  Not meeting the physical requirements such as handgrip force and gait speed.  Inability to maintain verbal communication with the study team.  Lack of physical independence.  Presence of severe neurological or cognitive impairments, significant cardiovascular diseases, major musculoskeletal deformities or injuries, or other chronic diseases.  Medication usage that might affect the assessments.  Having a Montreal Cognitive Assessment (MoCA) score below 26.  **Confirmed Eligible to Include in the Study:**  Out of the 45 eligible candidates, 42 were included in the study. The reasons for non-participation for the 3 individuals who dropped out were:Challenges related to adherence to the study's regimen ( personal issues, time constraints) |
|  |  | (c) Consider use of a flow diagram | Figure 1 |
| Descriptive data | 14* | (a) Give characteristics of study participants (eg demographic, clinical, social) and information on exposures and potential confounders | **Demographic Characteristics:**  **Age:**  Control group (CG): Average age = 81.1 ± 4.0 years.  Sarcopenic obese group (SOG): Average age = 77.7 ± 2.9 years.  **Body Mass Index (BMI, kg/m²):**  CG: BMI = 24.9 ± 0.6 kg/m².  SOG: BMI = 34.5 ± 3.2 kg/m².  **Gender:**  CG: M/F = 12/10  SOG: M/F = 12/8  **Potential Confounders:**  **Medication Usage:** Medications that might affect the assessments were considered as potential confounders. Individuals on such medications were excluded.  **Physical Independence:** This was a criterion for participation, suggesting that varying levels of physical independence might have affected the results.  **Health Status:** Based on the Questionnaire de Santé et Dossier Médical des Sujets, individual health histories, and other related health factors could be potential confounders.  **Physical Activity Level:** Assessed using the Ricci and Gagnon scales, this could play a significant role in influencing the outcomes of the assessments and act as a potential confounder.  **Cognitive Status:** As determined by the MoCA test, cognitive status might influence a participant's ability to understand and follow through with the instructions during the assessments. |
|  |  | (b) Indicate number of participants with missing data for each variable of interest | No missing data |
| Outcome data | 15* | Report numbers of outcome events or summary measures | **Anthropometric Measurements:**  Average height, waist circumference, and hip circumference.  Body mass (BM) and percentage of body fat mass (FBM, %).  Calculated values for fat body mass (FBM) and lean body mass (LBM).  **Static Steady-State Balance (Romberg Test):**  Average standing time in seconds (with maximum time being 30 seconds).  Number of terminations due to eye-opening, movements to regain stability, or needing assistance.  **Proactive Balance (FRT):**  Average maximal reach distance in centimeters.  **Timed Up and Go Test (TUG):**  Average time in seconds for participants to complete the TUG test.  **10-meter Walking Test:**  Gait speed  **Spatiotemporal gait parameters:**  Cadence (average strides/min).  Speed (average m/s).  Stride length (average cm).  Stride time (average seconds).  **Bilateral spatiotemporal parameters (expressed as a percentage of the gait cycle):**  Stance phase.  Swing phase.  1st double support.  Single support.  2nd double support. |
| Main results | 16 | (*a*) Give unadjusted estimates and, if applicable, confounder-adjusted estimates and their precision (eg, 95% confidence interval). Make clear which confounders were adjusted for and why they were included | Not applicable |
|  |  | (*b*) Report category boundaries when continuous variables were categorized | Not applicable |
|  |  | (*c*) If relevant, consider translating estimates of relative risk into absolute risk for a meaningful time period | Not applicable |
| Other analyses | 17 | Report other analyses done—eg analyses of subgroups and interactions, and sensitivity analyses | Not applicable |
| Discussion | | |  |
| Key results | 18 | Summarise key results with reference to study objectives | The study sought to investigate the effects of obesity on balance and walking characteristics among older adults with SO and understand the relationship between various anthropometric attributes and corresponding gait and balance measurements.  **Effect on Balance:** The study found that obesity significantly impacts steady-state and proactive balance attributes. A 20% decline in steady-state equilibrium was observed among the SO group using the Romberg Test. The Functional Reach Test (FRT) and Timed Up and Go Test (TUG) showed a 14% and 12.5% decline respectively.  **Effect on Gait:** The gait analysis revealed a decline in gait speed in the elderly with SO, characterized by extended step lengths and a longer support phase during walking, particularly the double support phase.  **Interrelation with Body Metrics:** While factors like body weight, BMI, and body fat play roles, LBM has the most substantial influence, especially in SO individuals. |
| Limitations | 19 | Discuss limitations of the study, taking into account sources of potential bias or imprecision. Discuss both direction and magnitude of any potential bias | **Sample Size:** The study involved a relatively small cohort of 42 subjects, making it challenging to generalize findings due to inherent variability and heterogeneity found in older populations.  **Measurement Tools:** The study utilized impedance-meters to determine LBM and FBM. These may not be as precise as other tools like Dual-energy X-ray Absorptiometry.  **Potential Bias:** Given the limited sample size, selection bias could be a concern, possibly affecting the magnitude of the results. If the participants were not representative of the broader older adults SO population, the results might over or under-estimate the actual relationship between obesity and gait/balance. |
| Interpretation | 20 | Give a cautious overall interpretation of results considering objectives, limitations, multiplicity of analyses, results from similar studies, and other relevant evidence | While the study found clear associations between obesity and gait and balance impairments in SO individuals, caution must be exercised due to its limitations. The consistent and strong relationship between LBM and gait and balance parameters, especially in those with SO, is noteworthy. This emphasizes the importance of muscle mass in ensuring stability and functional mobility. However, given the small sample size and potential measurement imprecisions, these findings should be seen as preliminary and exploratory.  When viewed in conjunction with other studies that have investigated obesity's impact on gait and balance (but not necessarily on SO populations), the results seem consistent with the broader literature |
| Generalisability | 21 | Discuss the generalisability (external validity) of the study results | The results offer valuable insights into the effects of obesity on elderly individuals diagnosed with SO. However, due to the limitations highlighted earlier, especially the small sample size, it's challenging to generalize these findings to the broader elderly SO population. The study sets a foundation for larger-scale investigations using more precise measurement tools to confirm and expand on these preliminary findings. It is essential for future research to address these limitations to provide more definitive conclusions that can be generalized more broadly. |
| Other information | | |  |
| Funding | 22 | Give the source of funding and the role of the funders for the present study and, if applicable, for the original study on which the present article is based | This research received no external funding. |
